# Oxidative Stress and Inflammation in Colorectal Cancer–Redox–Immune Crosstalk, Biomarkers, and Translational Implications: A Qualitative Systematic Review

**DOI:** 10.3390/life16030424

**Published:** 2026-03-05

**Authors:** Razvan Marinescu, Daniela Marinescu, Daniel Preda, Ana-Maria Ciurea, Petrica Popa, Lidia Boldeanu, Marius Bica, Mihai Boldeanu, Stefan Patrascu, Marin Valeriu Surlin

**Affiliations:** 1 Department of Surgery, Faculty of Medicine, University of Medicine and Pharmacy Craiova, Petru Rares 2-4, 200349 Craiova, Romania; razvanalexandrumarinescu98@gmail.com (R.M.); daniela.marinescu@umfcv.ro (D.M.); daniel.preda@umfcv.ro (D.P.); petrica.popa@umfcv.ro (P.P.); marius.bica@umfcv.ro (M.B.); mihail.boldeanu@umfcv.ro (M.B.); vsurlin@gmail.com (M.V.S.); 2 1st Clinic of Surgery, Clinical Emergency County Hospital Craiova, Tabaci 1st, 200642 Craiova, Romania; 3 Department of Oncology, “St Nectarie” Clinical Oncology Hospital of Craiova, Caracal 23, 200347 Craiova, Romania; 4 Clinic of Gastroenterology, Clinical Emergency County Hospital Craiova, Tabaci 1st, 200642 Craiova, Romania; 5 Department of Microbiology, University of Medicine and Pharmacy Craiova, Petru Rares 2-4, 200349 Craiova, Romania; lidia.boldeanu@umfcv.ro; 6 Department of Immunology, “Filantropia” Clinical Hospital, Filantropiei 1st, 200143 Craiova, Romania; 7 Academy of Romanian Scientists, Ilfov Street no. 3, 050044 Bucharest, Romania

**Keywords:** colorectal cancer, oxidative stress, inflammation, biomarkers, molecular pathways, redox signaling, therapy resistance, therapeutic strategies

## Abstract

**Highlights:**

- Oxidative stress and chronic inflammation cooperate to drive colorectal carcinogenesis, tumor progression, invasion, and therapy resistance.
- NF-κB, NRF2, and IL-6/JAK/STAT3 are central redox-sensitive signaling nodes with therapeutic relevance.
- Biomarkers (8-OHdG, MDA, F_2_-isoprostanes, CRP, IL-6, and TNF-α) may refine diagnosis, prognosis, and monitoring.
- Aspirin/COX-2 inhibition, antioxidants, and pathway modulators show preventive/adjunctive potential.
- Combinations with checkpoint inhibitors and microbiota modulation are emerging translational avenues.

**Abstract:**

Oxidative stress and chronic inflammation are tightly interconnected biological processes that play central roles in colorectal cancer (CRC) initiation, progression, and resistance to therapy. Understanding their reciprocal interactions may identify novel biomarkers and therapeutic targets with translational relevance. Methods: This systematic review with qualitative synthesis was conducted in accordance with the PRISMA 2020 guidelines. A comprehensive literature search of PubMed/MEDLINE, Scopus, Web of Science, and Google Scholar was performed from January 2005 to June 2025. Eligible studies investigated mechanistic links between oxidative stress and inflammation in colorectal cancer, assessed oxidative or inflammatory biomarkers, or explored redox- and inflammation-targeted therapeutic strategies. Study selection and data extraction were performed systematically. Due to heterogeneity in study designs and outcomes, a qualitative synthesis was undertaken without meta-analysis. Results: Twenty-six studies met the inclusion criteria. Evidence consistently demonstrated that redox-sensitive pathways—including NF-κB, NRF2, and IL-6/JAK/STAT3—drive colorectal carcinogenesis by promoting genomic instability, immune evasion, angiogenesis, and therapy resistance. Biomarkers such as 8-hydroxy-2′-deoxyguanosine, malondialdehyde, F_2_-isoprostanes, C-reactive protein, interleukin-6, and tumor necrosis factor-α were frequently associated with tumor stage, prognosis, and treatment response. Therapeutic strategies targeting oxidative stress and inflammation showed promising preclinical and early translational results, particularly in combination with chemotherapy or immunotherapy. Conclusion: Oxidative stress and inflammation constitute a synergistic axis that critically influences colorectal cancer biology. Although several biomarkers and redox-targeted interventions demonstrate translational potential, robust clinical validation is still required before routine implementation. Integrative strategies guided by biomarker profiling may represent a future direction for personalized CRC management. Most therapeutic approaches discussed are supported by preclinical and early translational evidence, and their clinical applicability remains to be validated.

## 1. Introduction

Rationale

Colorectal cancer (CRC) remains one of the most prevalent malignancies worldwide and a leading cause of cancer-related mortality. According to GLOBOCAN 2023, an estimated 1.9 million people were diagnosed with CRC in the world [1]. In 2019 alone, about 150,000 new cases and 52,000 deaths of CRC were recorded in the United States [2]. CRC is still one of the major causes of death from cancer in industrial countries.

Despite advances in surgical techniques and multimodal oncologic therapies, recurrence, metastasis, and therapeutic resistance continue to limit long-term survival, particularly in advanced disease stages [3]. The negative consequences, as well as the emergence of resistance to traditional therapies, are the two major limiting factors of their efficacy.

Therefore, there is a need for a deeper understanding of the pathogenesis of colorectal cancer and the development of new treatment methods and diagnostic tools [4].

Increasing evidence indicates that oxidative stress and chronic inflammation play pivotal roles in CRC initiation and progression by shaping the tumor microenvironment, altering cellular signaling, and promoting immune dysregulation.

Reactive oxygen species (ROS), while essential for physiological signaling, become pathogenic when produced in excess or inadequately neutralized. In parallel, persistent inflammatory signaling sustains ROS generation, establishing a self-perpetuating loop that favors malignant transformation.

Objectives

The objective of this systematic review was to qualitatively synthesize current evidence on the reciprocal interplay between oxidative stress and chronic inflammation in colorectal cancer, with a specific focus on redox-sensitive molecular pathways involved in carcinogenesis and tumor progression, clinically relevant oxidative and inflammatory biomarkers, and translational therapeutic implications, including mechanisms of therapy resistance.

## 2. Methods

This systematic review was conducted in accordance with the Preferred Reporting Items for Systematic Reviews and Meta-Analyses (PRISMA) 2020 guidelines, using a PICo (Population, Phenomenon of Interest, Context) qualitative framework.

Protocol and registration

The review protocol was not prospectively registered in a publicly accessible database such as PROSPERO. However, the research objectives, eligibility criteria, and qualitative synthesis approach were clearly defined before initiating the literature search and were maintained throughout the review process. While prospective registration would have further strengthened methodological transparency, the predefined framework was followed consistently during study selection, data extraction, and synthesis.

Eligibility criteria

Studies were eligible for inclusion if they met the following criteria: (1) investigated mechanistic links between oxidative stress and chronic inflammation in colorectal cancer; (2) evaluated oxidative or inflammatory biomarkers with diagnostic, prognostic, or predictive relevance; or (3) explored redox- or inflammation-targeted therapeutic strategies in preclinical, translational, or clinical settings.

Eligible study designs included observational clinical studies, translational human studies, and relevant mechanistic investigations. Reviews, editorials, conference abstracts without full peer-reviewed data, non-English publications, outdated studies, and reports lacking sufficient methodological detail were excluded.

For qualitative synthesis, included studies were grouped according to their primary focus into three categories: mechanistic pathway studies, biomarker-based investigations, and translational or therapeutic studies.

Narrative reviews and meta-analyses were used to provide mechanistic and contextual background and were not considered sources of primary outcome data for the qualitative synthesis.

Information sources

A comprehensive literature search was performed in the PubMed/MEDLINE, Scopus, Web of Science, and Google Scholar databases, covering publications from January 2005 to June 2025. In addition, the reference lists of included articles were manually screened to identify potentially relevant studies. The final search of all databases was conducted on 30 June 2025.

Search strategy

The search strategy was developed using a combination of Medical Subject Headings (MeSH) and free-text terms related to colorectal cancer, oxidative stress, and inflammation. The PubMed/MEDLINE search strategy was as follows:

(“colorectal cancer” [MeSH] OR “colon cancer” OR “rectal cancer”) AND (“oxidative stress” [MeSH] OR “reactive oxygen species” OR ROS) AND (“inflammation” [MeSH] OR “inflammatory signaling” OR cytokines).

Filters applied included the English language and publication dates from 1 January 2005 to 30 June 2025. Similar search strategies, adapted to the specific syntax of each database, were applied to Scopus, Web of Science, and Google Scholar (Supplementary Material S1).

Data collection process

Data were extracted independently by two reviewers using a predefined data extraction form. Extracted information included study characteristics, investigated molecular pathways, oxidative and inflammatory biomarkers, clinical correlations, and therapeutic implications. Any discrepancies in data extraction were resolved through discussion and consensus. When necessary, original study reports were re-examined to clarify unclear or missing information. No automation tools were used in the data collection process.

Data items

The primary outcomes of interest were qualitative evidence describing the mechanistic interplay between oxidative stress and chronic inflammation in colorectal cancer, including redox-sensitive signaling pathways involved in carcinogenesis, tumor progression, and therapy resistance. Secondary outcomes included the identification and clinical relevance of oxidative and inflammatory biomarkers, as well as reported translational and therapeutic implications.

Additional variables extracted from each study included study design, study population or experimental model, type of biological material analyzed, investigated molecular pathways or biomarkers, and reported clinical correlations. When information was incomplete or unclear, data were interpreted as reported without imputation.

Study Risk-of-Bias Assessment

Given the diversity of study designs included in this review—ranging from observational clinical studies to experimental preclinical models—we adopted a design-specific approach to bias appraisal. Observational clinical studies were evaluated using the Newcastle–Ottawa Scale (NOS), which assesses selection, comparability, and outcome domains.

Preclinical investigations were appraised using criteria adapted from the SYRCLE risk-of-bias framework, focusing on allocation methods, reporting transparency, definition of outcomes, and reproducibility of experimental conditions.

In view of the qualitative nature of this systematic review and the heterogeneity of included study designs, these tools were applied descriptively to identify methodological strengths and limitations rather than to generate aggregated numerical scores.

Narrative reviews and meta-analyses were not subjected to formal bias assessment, as they were used to provide contextual and mechanistic background rather than primary outcome data.

All assessments were performed independently by two reviewers, and discrepancies were resolved through discussion.

Synthesis methods

Studies eligible for qualitative synthesis were selected according to the predefined eligibility criteria and grouped based on their primary focus, including mechanistic pathway analyses, biomarker-based investigations, and translational or therapeutic studies.

Data were synthesized through thematic narrative summarization. Key findings were extracted and organized around recurring molecular pathways, oxidative and inflammatory biomarkers, and their reported clinical or experimental implications. No data conversions or imputations were required.

Given the substantial heterogeneity in study design, patient populations, experimental models, and outcome measures, quantitative synthesis and meta-analysis were not considered appropriate. Consequently, formal statistical assessments of heterogeneity, subgroup analyses, or meta-regression were not performed. As no quantitative synthesis or pooled effect estimation was performed, effect measures were not predefined.

The overall interpretation of the findings was informed by study design and structured risk-of-bias appraisal rather than by formal GRADE methodology, which was not applied due to the qualitative nature of the synthesis.

## 3. Results

### 3.1. Study Selection

**Figure 1:**
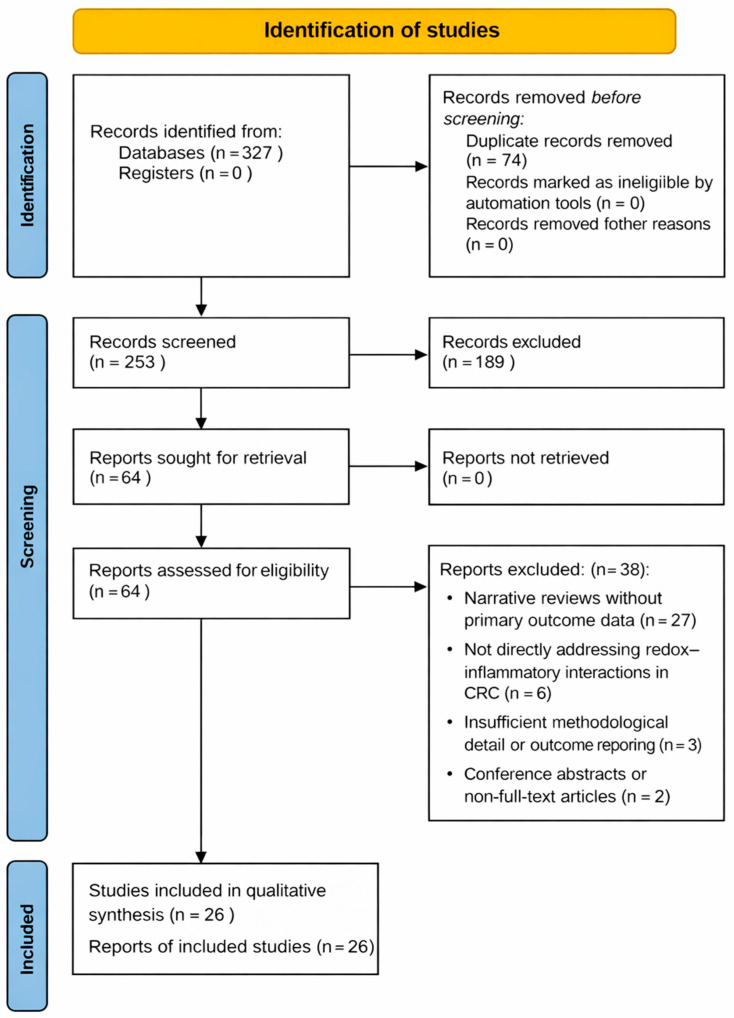
The PRISMA 2020 flow diagram for the identification of studies.

The database search identified 327 records. After duplicate removal, 64 articles were retrieved and assessed at the full-text level. Following full-text evaluation, 26 studies met the predefined inclusion criteria and were included in the qualitative synthesis. An overview of the study selection process is presented in Figure 1.

### 3.2. Study Characteristics

An initial total of 327 records was identified through database searches. After the removal of duplicate records, titles and abstracts were screened independently by two reviewers to assess eligibility. Full-text articles of potentially relevant studies were subsequently retrieved and evaluated independently by the same two reviewers according to the predefined inclusion and exclusion criteria. Any disagreements were resolved through discussion and consensus.

Sixty-four articles were assessed for full-text eligibility. Of these, 26 primary clinical and experimental studies met the predefined inclusion criteria and were included in the qualitative synthesis. The characteristics of these studies are summarized in Table 1 and Table 2. Narrative reviews and theoretical articles were excluded from the primary qualitative synthesis but were retained as contextual references and are summarized separately in Table 3.

Additional references cited throughout the manuscript were used exclusively to support epidemiological background and mechanistic interpretation and were not considered eligible for qualitative synthesis.

The study selection process is illustrated in Figure 1. No automation tools were used during the study selection process.

### 3.3. Results of Individual Studies

Across the 26 included primary studies, consistent qualitative patterns were observed. The most representative mechanistic and translational findings are summarized below.

Experimental investigations demonstrated that NRF2 signaling plays a dual but predominantly tumor-promoting role in established CRC. Huang et al. showed that inhibition of NRF2 increased chemosensitivity by promoting ferroptosis and pyroptosis in CRC models [15]. Similarly, Evans et al. reported that pharmacologic inhibition of NRF2 using brusatol suppressed tumor growth in vivo [16].

ROS-driven activation of pro-survival signaling cascades was repeatedly observed. Dong et al. demonstrated that ROS-mediated activation of the PI3K/AKT and Wnt/β-catenin pathways induced HIF-1α–dependent metabolic reprogramming and 5-fluorouracil resistance [17]. Wang T. et al. further showed that IL-6–mediated STAT3 activation promoted epithelial–mesenchymal transition and an aggressive tumor phenotype in CRC models [18].

Angiogenic remodeling was closely linked to oxidative signaling. Manuelli et al. identified NOX4-derived ROS as a driver of HIF-1α stabilization and angiogenesis in CRC models [19]. Zhou et al. reported that inhibition of HIF-1α signaling reduced VEGF expression and suppressed angiogenesis [20].

Translational cohort data provided clinical corroboration of redox-driven resistance. O’Cathail et al. demonstrated that high NRF2 transcriptional activity predicted a poor response to chemoradiation in rectal cancer patients [5].

Collectively, these findings indicate convergence of oxidative stress and inflammatory signaling on the NRF2, STAT3, NF-κB, and HIF-1α axes, promoting tumor progression and resistance phenotypes.

### 3.4. Biomarkers of Oxidative Stress and Inflammation in Colorectal Cancer

Oxidative Stress Biomarkers

Observational clinical studies consistently reported associations between oxidative damage markers and colorectal cancer progression. Kang et al. demonstrated that elevated tumoral 8-hydroxy-2′-deoxyguanosine (8-OHdG) expression was significantly associated with reduced overall survival in CRC patients [6].

In a case–control study, Acevedo-León et al. reported significantly higher circulating levels of 8-OHdG and F_2_-isoprostanes in CRC patients compared with controls, with concentrations correlating with advanced tumor stage [7].

Rašić et al. observed progressive increases in serum malondialdehyde (MDA) levels across CRC stages, supporting a stage-dependent association between lipid peroxidation and tumor burden [8].

Mariani et al. demonstrated the detectability of oxidative stress markers in liquid biopsy samples, supporting the feasibility of minimally invasive monitoring approaches [9].

Inflammatory Biomarkers

Systemic inflammatory markers were also associated with CRC prognosis. A pooled meta-analysis by Partl et al. identified elevated C-reactive protein (CRP) as an adverse prognostic factor in colorectal cancer [10].

Experimental and translational evidence further implicated IL-6-driven STAT3 activation in tumor aggressiveness, as demonstrated by Wang T. et al. [18].

Together, these findings support the clinical relevance of systemic inflammation as both a prognostic indicator and a mechanistic contributor to CRC progression.

Combined Biomarker Approaches

Combined assessment of oxidative and inflammatory biomarkers appeared to enhance prognostic discrimination. Acevedo-León et al. reported stronger correlations with tumor stage when oxidative stress markers were evaluated alongside inflammatory mediators [7].

However, variability in analytical methods, absence of standardized cut-off values, and potential systemic confounders were recurrent methodological limitations across clinical studies [6,7,8].

### 3.5. Results of Synthesis

Overall, the integration of primary clinical and experimental studies revealed consistent qualitative evidence supporting a central role for oxidative stress and chronic inflammation in colorectal cancer progression and treatment resistance. Observational clinical studies, evaluated using the Newcastle–Ottawa Scale (NOS), demonstrated generally acceptable methodological quality in domains such as case definition and outcome assessment. However, most were retrospective in design, involved relatively small cohorts, and lacked predefined biomarker thresholds or comprehensive adjustment for confounders, which limited internal validity and the strength of causal inference.

Preclinical investigations, appraised using criteria adapted from the SYRCLE framework, consistently confirmed activation of oxidative stress-activated pathways, including NRF2, STAT3, HIF-1α, PI3K/AKT, and Wnt/β-catenin. Replication of these mechanisms across independent experimental models reinforced biological plausibility. Nevertheless, reporting of allocation methods, randomization procedures, and blinding was frequently incomplete, and external validation across laboratories remained limited.

Although mechanistic convergence across studies was notable, heterogeneity in clinical design, patient populations, biomarker measurement techniques, and outcome definitions precluded quantitative synthesis. Taken together, the evidence supports a coherent redox–inflammatory axis in CRC biology. From a methodological perspective, clinical findings provide low-to-moderate certainty of evidence, primarily due to design-related constraints, whereas preclinical mechanistic data offer moderate confidence based on reproducible pathway activation. Further well-designed prospective human studies are required to strengthen clinical certainty and enable reliable translational application.

## 4. Discussion

### 4.1. Oxidative Stress and Inflammation in Colorectal Carcinogenesis

This systematic review highlights the central role of the redox–inflammatory interplay in colorectal cancer (CRC) biology. Oxidative stress results from the excessive production of reactive oxygen species (ROS) that overwhelms cellular antioxidant defense systems, leading to oxidative injury in gastrointestinal epithelial cells, including DNA damage, protein denaturation, and lipid peroxidation, which collectively disrupt cellular homeostasis [50].

When ROS interact with critical macromolecules such as DNA, proteins, and lipids, they induce base modifications as well as single- and double-strand breaks, resulting in the formation of apurinic/apyrimidinic sites and increased genomic instability [52]. Oxidative stress-induced mucosal injury triggers inflammatory repair responses; however, when inflammation becomes chronic, epithelial regeneration is impaired, mucosal barrier integrity is compromised, and autoimmune-like reactions may develop [51]. Chronic inflammation further amplifies ROS production, establishing a self-perpetuating cycle of oxidative stress and inflammatory signaling. This feedback loop activates redox-sensitive pathways, including NF-κB and STAT3, promotes secretion of pro-inflammatory cytokines such as IL-6 and TNF-α, and induces epigenetic and metabolic alterations that facilitate angiogenesis and accelerate colorectal tumor development [53]. Once established, colorectal tumors further reinforce this cycle through sustained release of inflammatory mediators.

The pathogenetic relevance of this interplay is exemplified in inflammatory bowel disease (IBD), where persistent immune activation and redox imbalance in ulcerative colitis and Crohn’s disease create a pro-carcinogenic intestinal environment [54]. Moreover, several genetic loci related to oxidative stress regulation have been linked to increased susceptibility to IBD, supporting the concept that chronic oxidative inflammation primes intestinal epithelial cells for malignant transformation through redox-dependent activation of transcriptional and signaling pathways [27].

At the molecular level, oxidative stress and chronic inflammation interact through multiple signaling cascades that orchestrate colorectal carcinogenesis by influencing cellular redox balance, gene expression, and the tumor microenvironment. ROS function not only as damaging agents but also as secondary messengers that modulate pathways controlling proliferation, apoptosis, angiogenesis, and immune evasion. Persistent activation of these redox-sensitive networks drives genomic instability, aberrant transcriptional activity, and metabolic reprogramming, contributing to CRC initiation and progression [53].

#### 4.1.1. NF-κB Signaling Pathway

The nuclear factor kappa-B (NF-κB) pathway represents a central molecular link between inflammation and colorectal carcinogenesis. Under oxidative stress, ROS activate the IκB kinase complex, leading to the phosphorylation and degradation of IκBα and subsequent nuclear translocation of NF-κB. Activated NF-κB induces transcription of genes encoding pro-inflammatory cytokines (IL-6, TNF-α), anti-apoptotic proteins (Bcl-2, XIAP), and enzymes such as COX-2 and iNOS, thereby promoting tumor cell survival, proliferation, angiogenesis, immune evasion, and resistance to apoptosis [28,29]. Persistent NF-κB activation sustains a pro-inflammatory tumor microenvironment and contributes to resistance phenotypes.

#### 4.1.2. NRF2 Pathway

The nuclear factor erythroid 2–related factor 2 (NRF2) pathway constitutes the primary cellular defense mechanism against oxidative stress. Under physiological conditions, NRF2 is sequestered in the cytoplasm by Keap1 and targeted for proteasomal degradation. Elevated ROS levels or electrophilic stress modify Keap1 cysteine residues, allowing for NRF2 nuclear translocation and induction of antioxidant and cytoprotective enzymes such as GPx, SOD, HO-1, and NQO1. While transient NRF2 activation preserves genomic stability and protects against carcinogenesis, sustained NRF2 upregulation in established CRC promotes metabolic adaptation, enhanced antioxidant capacity, chemoresistance, and tumor progression [30].

#### 4.1.3. STAT3 Signaling Pathway

STAT3 is a redox-sensitive transcription factor activated by cytokines such as IL-6 and by ROS. Following phosphorylation, STAT3 dimerizes and translocates to the nucleus, inducing genes involved in proliferation (cyclin D1), survival (Bcl-xL), angiogenesis, and immune evasion (PD-L1). Persistent STAT3 activation supports epithelial–mesenchymal transition, angiogenesis, and immune suppression, thereby sustaining colorectal tumor growth and resistance to therapy [31].

#### 4.1.4. Oxidative Stress-Mediated Epigenetic Alterations

Oxidative stress also contributes to colorectal carcinogenesis through epigenetic reprogramming. ROS activate DNA methyltransferases, leading to hypermethylation and silencing of tumor suppressor genes such as MLH1 and CDKN2A. In addition, ROS modify histone acetylation and methylation patterns and regulate non-coding RNA expression, including microRNAs and long non-coding RNAs, thereby reinforcing malignant transformation and tumor progression [32].

### 4.2. Role of Oxidative Stress in Tumor Progression and Microenvironmental Remodeling

Although oxidative stress is critical for tumor initiation, its impact becomes particularly evident during CRC progression. In established tumors, sustained redox imbalance actively influences tumor behavior, cellular adaptation, and interactions with the surrounding microenvironment.

The tumor microenvironment (TME) plays a central role in shaping redox balance during CRC progression. In addition to malignant epithelial cells, stromal components such as cancer-associated fibroblasts, endothelial cells, macrophages, and neutrophils are major sources of ROS. The combined activity of these cell populations generates a persistently oxidative and inflammatory milieu that promotes tumor growth, invasion, and immune evasion [33].

Elevated ROS levels within the TME activate signaling pathways that enhance phenotypic plasticity and tumor adaptability. Oxidative stress facilitates epithelial–mesenchymal transition, increases migratory capacity, and supports survival under hypoxic and nutrient-limited conditions. At the same time, redox signaling modulates immune cell function, favoring immunosuppressive populations and impairing effective antitumor immune responses [33].

ROS stabilize hypoxia-inducible factor-1α and promote secretion of vascular endothelial growth factor and matrix metalloproteinases, thereby enhancing angiogenesis, extracellular matrix remodeling, and metastatic dissemination. Hypoxia, oxidative stress, and chronic inflammation reinforce one another in a self-sustaining feedback loop that drives an invasive, angiogenic, and immunosuppressive tumor phenotype [55]. Metabolic reprogramming represents a key adaptive response to oxidative stress, with activation of pathways such as Wnt/β-catenin, PI3K/AKT, and KRAS supporting glycolytic metabolism, altered lipid utilization, and increased antioxidant capacity [55].

### 4.3. Angiogenesis, Invasion, and Metastasis

Redox signaling plays a pivotal role in angiogenesis, which is essential for tumor growth and metastasis. In CRC, ROS stabilize hypoxia-inducible factor-1α under hypoxic conditions, leading to increased VEGF expression and endothelial cell proliferation [19]. Experimental NRF2 knockdown models demonstrate that weakened antioxidant defenses increase ROS levels and further stabilize HIF-1α, thereby promoting angiogenesis in vivo [34]. In contrast, pharmacologic agents such as Tanshinone IIA suppress HIF-1α and VEGF signaling and inhibit angiogenesis in CRC models [20].

ROS also facilitate invasion and metastasis by upregulating matrix metalloproteinases, particularly MMP-2 and MMP-9, which degrade extracellular matrix components and promote tumor cell migration. In addition, ROS enhance vasculogenic mimicry through HIF-1α-dependent mechanisms, allowing cancer cells to form vessel-like structures that support tumor perfusion. Inhibition of ROS with N-acetylcysteine reduces MMP expression and disrupts vasculogenic mimicry, confirming their role in metastatic progression [20].

### 4.4. Oxidative Stress and Therapy Resistance

Despite advances in multimodal CRC treatment, therapeutic resistance remains a major obstacle to durable disease control. Increasing evidence implicates the redox-driven inflammatory axis as a central driver of resistance to chemotherapy, radiotherapy, targeted agents, and immunotherapy [35].

Chronic oxidative stress induces sustained activation of NRF2, leading to upregulation of antioxidant enzymes and glutathione biosynthesis, which neutralize therapy-induced ROS and reduce cytotoxic efficacy [36,56]. High NRF2 activity correlates with poor response to 5-fluorouracil- and oxaliplatin-based regimens [15]. Concurrent activation of HIF-1α and NF-κB promotes epithelial–mesenchymal transition, anti-apoptotic signaling, and metabolic reprogramming, further enhancing resistance [17,18].

In radiotherapy, NRF2 activation and hypoxia-mediated HIF-1α stabilization attenuate ROS-induced DNA damage, while cytokine-driven STAT3 and NF-κB signaling promote survival and repair responses that confer radioresistance [5,37,38,39]. Moreover, ROS-dependent activation of PI3K/AKT and MAPK pathways and increased PD-L1 expression contribute to resistance to EGFR-targeted therapies and immune checkpoint inhibitors [40,41,57].

Cancer stem cells represent an additional redox-adapted population that contributes to therapeutic failure. These cells maintain low intracellular ROS levels through enhanced antioxidant capacity and NRF2 signaling, enabling survival under cytotoxic stress and promoting minimal residual disease and recurrence [42,43].

### 4.5. Biomarkers of Oxidative Stress and Inflammation: Translational Relevance

Across clinical studies, biomarkers of oxidative DNA damage, lipid peroxidation, and inflammatory signaling are consistently elevated in CRC patients compared with healthy controls and are frequently associated with advanced disease stage and poorer clinical outcomes. Markers such as 8-hydroxy-2′-deoxyguanosine, malondialdehyde, F_2_-isoprostanes, and nitric oxide metabolites reflect cumulative oxidative damage and redox imbalance [6,7,8,43,58,59]. Similarly, inflammatory biomarkers, including C-reactive protein, IL-6, IL-8, and TNF-α, indicate chronic activation of inflammatory pathways closely linked to tumor progression and immune modulation [10,44,45,60].

Several studies suggest that a combined assessment of oxidative stress and inflammatory biomarkers may improve diagnostic and prognostic performance compared with individual markers. When integrated with established tumor markers such as CEA and CA19-9, these composite panels may enhance patient stratification and monitoring during treatment [9]. However, variability in analytical methods and the lack of standardized cut-off values currently limit their routine clinical application.

Beyond methodological variability, broader translational challenges remain. Most oxidative stress biomarkers lack analytical standardization, and validated clinical thresholds are rarely established. Differences in assay methodology, sample processing, and biological matrices—such as serum, plasma, tissue, or urine—introduce variability that complicates inter-study comparability.

Moreover, many of these biomarkers reflect systemic oxidative burden rather than tumor-specific processes, reducing their specificity for colorectal cancer. The influence of comorbid conditions, including chronic inflammatory disorders, metabolic syndrome, or smoking status, may further confound interpretation.

Although the biological plausibility of the redox–inflammatory axis in CRC is well supported, translation into routine clinical practice requires prospective validation, standardized analytical protocols, and clearly defined diagnostic or prognostic thresholds.

### 4.6. Therapeutic and Translational Implications

Recognition of the interconnected roles of oxidative stress and inflammation has driven the development of therapeutic strategies aimed at modulating redox balance and inflammatory signaling. These include antioxidant-based interventions, pro-oxidant therapies, anti-inflammatory agents, and targeted modulation of redox-sensitive pathways, often in combination with standard oncologic treatments [11,12,22,24,61].

Anti-inflammatory approaches such as long-term aspirin use and selective COX-2 inhibition reduce CRC incidence and adenoma burden but require careful risk–benefit assessment [13,14]. Targeted cytokine inhibition, including IL-6 receptor blockade, attenuates STAT3 activation and tumor vascularization in preclinical models [25]. Therapeutic targeting of NF-κB, NRF2, and JAK/STAT3 signaling has shown promise in overcoming resistance, although context-dependent effects necessitate precise therapeutic modulation [16,26,46,62].

### 4.7. Integrative Strategies and Future Perspectives

Emerging integrative approaches include combination regimens that exploit synergistic interactions between redox modulation and immunotherapy. High-dose pharmacologic vitamin C enhances immunogenic cell death and potentiates immune checkpoint blockade, while polyphenols such as epigallocatechin gallate modulate PD-L1 expression and immune cell infiltration [21,47,63,64]. Modulation of the gut microbiota through dietary interventions, prebiotics, and probiotics may further support intestinal redox balance and suppress inflammatory cytokine production [48,49].

Integration of oxidative stress and inflammatory biomarkers with liquid biopsy technologies, multi-omics profiling, and artificial intelligence-driven predictive models may enable identification of patient subgroups most likely to benefit from redox- and inflammation-targeted therapies, supporting the transition toward precision oncology in CRC.

### 4.8. Limitations

Several limitations of this review should be acknowledged.

First, the protocol was not prospectively registered (e.g., in PROSPERO). Although the objectives and methodological approach were clearly defined before the literature search and consistently followed throughout the review process, prospective registration would have further strengthened transparency and reproducibility. In this context, the absence of registration may introduce a theoretical risk of selection or reporting bias at the review level.

Second, the heterogeneity of the included studies—both in design and in biomarker assessment—limits direct comparability across findings and precludes quantitative synthesis. Although structured risk-of-bias tools were applied to observational and preclinical studies, variability in methodology and outcome reporting reduces overall certainty.

Third, most oxidative stress biomarkers are not yet standardized for routine clinical use. Differences in assay methodology, biological matrices, and the influence of systemic inflammatory or metabolic comorbidities complicate interpretation and reduce specificity for tumor-related processes.

Finally, the predominance of preclinical experimental models limits immediate translational applicability. While mechanistic findings were largely consistent, large prospective and well-controlled clinical studies are necessary before redox- or inflammation-based strategies can be reliably implemented in routine CRC management.

Publication bias cannot be excluded, as studies reporting positive associations between oxidative stress, inflammation, and colorectal cancer biology may be overrepresented in the literature.

## 5. Conclusions

Recent findings indicate that the redox-inflammatory axis not only plays a central role in carcinogenesis but may also offer meaningful opportunities for colorectal cancer prevention and treatment. At the same time, the complexity of redox signaling and its strong dependence on biological context highlight the need for well-designed studies that go beyond purely descriptive analyses. Although emerging biomarkers and redox-targeted therapeutic strategies are encouraging, their translation into clinical practice is currently limited by substantial heterogeneity in study design, analytical methods, and patient populations. Increasing evidence suggests that combining multiple biomarkers, rather than relying on single markers, together with carefully controlled modulation of redox and inflammatory pathways, may provide greater clinical relevance. Ultimately, translation into routine clinical practice will depend on confirmation in rigorously designed prospective studies capable of defining optimal intervention windows, minimizing unintended tumor adaptation, and clarifying the role of redox-based strategies within personalized colorectal cancer management.

## Figures and Tables

**Table 1:** Clinical observational and translational studies included in the qualitative synthesis (n = 11).

Author (Ref)	Year	Study Design	Population	Biomarker/Intervention	Main Findings	Clinical Implication
O’Cathail et al. [5]	2021	Cohort + translational	Rectal cancer (n = 127)	NRF2 activity	High NRF2 predicts poor response	Predictive biomarker
Kang et al. [6]	2023	Retrospective cohort	CRC patients	8-OHdG	Associated with reduced survival	Prognostic marker
Acevedo-León et al. [7]	2022	Case–control	CRC vs. controls	8-OHdG, F2-isoprostanes	Correlate with tumor stage	Diagnostic utility
Rašić et al. [8]	2021	Cross-sectional	CRC patients	MDA	Increases with stage	Disease progression marker
Mariani et al. [9]	2021	Translational	CRC plasma	Oxidative markers	Detectable in liquid biopsy	Non-invasive monitoring
Partl et al. [10]	2017	Meta-analysis	CRC patients	CRP	Associated with poor prognosis	Inflammatory marker
Dhillon et al. [11]	2010	Phase II trial	Advanced cancer	Curcumin	Modulates inflammatory signaling	Adjunct strategy
Patel et al. [12]	2010	Clinical trial	CRC patients	Resveratrol	Modulates proliferation markers	Translational antioxidant
Rothwell et al. [13]	2011	Meta-analysis	General population	Aspirin	Reduced CRC mortality	Chemoprevention
Steinbach et al. [14]	2000	RCT	Adenoma patients	Celecoxib	Reduced adenoma burden	COX-2 chemoprevention

Primary human studies providing clinical outcome data on oxidative stress, inflammatory biomarkers, redox pathways, or therapeutic interventions in colorectal cancer.

**Table 2:** Experimental and preclinical studies included in the qualitative synthesis (n = 13).

Author (Ref)	Year	Model	Pathway/Biomarker	Main Mechanistic Finding	Translational Relevance
Huang et al. [15]	2023	In vitro & in vivo	NRF2, ferroptosis, pyroptosis	NRF2 inhibition increases chemosensitivity via ferroptosis and pyroptosis	Targeting redox resistance mechanisms
Evans et al. [16]	2018	In vivo	Brusatol, NRF2	NRF2 inhibition suppresses CRC growth	Targeting antioxidant defenses
Dong et al. [17]	2022	In vitro	ROS/PI3K/AKT/Wnt/β-catenin	ROS-driven signaling induces 5-FU resistance via metabolic reprogramming	Mechanism of chemoresistance
Wang T. et al. [18]	2019	In vitro	IL-6/STAT3	IL-6 induces EMT and aggressive CRC phenotype	Inflammatory signaling target
Manuelli et al. [19]	2022	In vitro & in vivo	NOX4, ROS, HIF-1α	NOX4-derived ROS stabilize HIF-1α and promote angiogenesis	Anti-angiogenic targeting
Zhou et al. [20]	2020	In vitro & in vivo	HIF-1α, VEGF	Tanshinone IIA inhibits angiogenesis	Anti-angiogenic therapeutic compound
Zhao et al. [21]	2020	In vivo	EGCG + anti-CTLA-4	EGCG enhances immunotherapy efficacy	Combination redox-immunotherapy
Wang Y. et al. [22]	2021	In vivo	Bardoxolone, NRF2	NRF2 modulation suppresses tumor progression	Pharmacologic NRF2 targeting
Catalano et al. [23]	2021	In vitro	ROS/Wnt/β-catenin	Oxidative stress modulates Wnt signaling	Redox-mediated tumor progression
Siddiqui et al. [24]	2015	In vitro	EGCG	Sensitizes CRC cells to 5-FU	Adjunct chemotherapy strategy
Chang et al. [25]	2015	In vivo	Metabolic competition	Tumor microenvironment metabolic stress drives progression	Microenvironmental targeting
Peng et al. [26]	2015	In vitro	Brusatol/NRF2	NRF2 inhibition sensitizes cancer cells to chemotherapy	Overcoming chemoresistance
Seril et al. [27]	2003	In vivo	ROS (colitis-associated model)	Oxidative stress promotes colitis-associated carcinogenesis	Inflammation-driven tumor initiation

Primary in vitro and in vivo studies investigating mechanistic interactions between oxidative stress and inflammation and their therapeutic implications in colorectal cancer.

**Table 3:** Narrative reviews and contextual secondary literature (n = 27).

Author (Ref)	Year	Type	Main Focus	Relevance to Current Review
Siegel R.L. [2]	2023	Epidemiological analysis	CRC statistics	Incidence and mortality context
Karin M. [28]	2005	Narrative review	NF-κB	Inflammation–cancer link
Grivennikov S.I. [29]	2010	Narrative review	NF-κB/STAT3	Inflammatory carcinogenesis
Rojo de la Vega M. [30]	2018	Narrative review	NRF2 hallmarks	Cancer redox biology
Johnson D.E. [31]	2018	Narrative review	IL-6/JAK/STAT3	Therapeutic targeting
Herceg Z. [32]	2011	Narrative review	Epigenetics & ROS	Redox epigenetic regulation
Kalyanaraman B. [33]	2017	Narrative review	Redox biology	Redox signaling framework
Kohan R. [34]	2020	Narrative review	ROS paradox	Tumor-promoting vs. suppressive roles
Wang M. et al. [35]	2023	Narrative review	ROS & chemoresistance	Redox-mediated therapy resistance
Hu M. et al. [36]	2024	Narrative review	NRF2 dual role	Context-dependent redox signaling
Bouleftour W. [37]	2021	Narrative review	Hypoxia & radioresistance	Radiation response modulation
Beckers C. et al. [38]	2024	Narrative review	Hypoxia & ROS	Radiotherapy resistance
Wang Q. et al. [39]	2022	Narrative review	Oxidative stress & drug resistance	Chemoresistance mechanisms
Antonangeli F. [40]	2020	Narrative review	PD-L1/NF-κB	Immune checkpoint regulation
Shan J. et al. [41]	2022	Narrative review	Immunotherapy resistance	Immune escape pathways
Hallis S.P. et al. [42]	2023	Narrative review	NRF2 & CSCs	Stem cell redox adaptation
McMillan D.C. [43]	2009	Narrative review	Systemic inflammation	Prognostic inflammation markers
Kumari N. [44]	2016	Narrative review	IL-6 signaling	Tumor-promoting inflammation
Balkwill F. [45]	2009	Narrative review	TNF-α	Tumor-promoting inflammation
Yu B. et al. [46]	2024	Narrative review	Immunotherapy	Checkpoint resistance mechanisms
Rodrigues J.A. [47]	2023	Narrative review	Photodynamic therapy	ROS-based translational strategies
O’Keefe S.J.D. [48]	2016	Narrative review	Microbiota	Diet–microbiome–CRC link
Schwabe R.F. & Jobin C. [49]	2013	Narrative review	Microbiome & inflammation	Cancer microenvironment
Perše M. [50]	2013	Narrative review	Oxidative stress	CRC pathogenesis
Reuter S. [51]	2010	Narrative review	ROS & inflammation	Mechanistic link
Catalano T. [52]	2025	Narrative review	ROS and theranostics	Central role of ROS in CRC
Li Q. et al. [53]	2024	Narrative review	NF-κB, STAT3 signaling	Inflammation-driven CRC pathways

Secondary literature used for conceptual and contextual background; these publications did not contribute primary outcome data to the qualitative synthesis.

## Data Availability

No additional data need to be published.

## Supplementary Materials

The following supporting information can be downloaded at: https://www.mdpi.com/article/10.3390/life16030424/s1, Supplementary S1: Search strategies.

## Abbreviations

The following abbreviations are used in this manuscript:

8-OHdG	8-hydroxy-2′-deoxyguanosine
5-FU	5-fluorouracil
AP	apurinic/apyrimidinic
ARE	antioxidant response element
bFGF	basic fibroblast growth factor
CA19-9	carbohydrate antigen 19-9
CDKN2A	cyclin-dependent kinase inhibitor 2A
CEA	carcinoembryonic antigen
COX-1	cyclooxygenase-1
COX-2	cyclooxygenase-2
CRC	colorectal cancer
CRP	C-reactive protein
CSC	cancer stem cell
DNA	deoxyribonucleic acid
EGCG	epigallocatechin gallate
EGFR	epidermal growth factor receptor
ELISA	enzyme-linked immunosorbent assay
EMT	epithelial–mesenchymal transition
ERK	extracellular signal-regulated kinase
F2-isoprostanes	F2-isoprostanes
GPx	glutathione peroxidase
HIF-1α	hypoxia-inducible factor 1-alpha
HO-1	heme oxygenase-1
IBD	inflammatory bowel disease
IKK	IκB kinase
IL	interleukin
iNOS	inducible nitric oxide synthase
JAK	Janus kinase
KRAS	Kirsten rat sarcoma viral oncogene homolog
MAPK	mitogen-activated protein kinase
MDA	Malondialdehyde
MeSH	Medical Subject Headings
MLH1	mutL homolog 1
MMP	matrix metalloproteinase
NAC	N-acetylcysteine
NF-κB	nuclear factor kappa B
NOx	nitric oxide metabolites
NQO1	NAD(P)H:quinone oxidoreductase 1
NRF2	nuclear factor erythroid 2–related factor 2
NSAIDs	nonsteroidal anti-inflammatory drugs
OS	oxidative stress
PD-1	programmed cell death protein 1
PD-L1	programmed death-ligand 1
PI3K	phosphoinositide 3-kinase
PRISMA	Preferred Reporting Items for Systematic Reviews and Meta-Analyses
ROS	reactive oxygen species
SIRT1	sirtuin 1
SOD	superoxide dismutase
STAT3	signal transducer and activator of transcription 3
TBARS	thiobarbituric acid-reactive substances
TME	tumor microenvironment
TNF-α	tumor necrosis factor alpha
VEGF	vascular endothelial growth factor
VM	vasculogenic mimicry
Wnt	wingless-related integration site
